# Dietary Fatty Acids and Inflammation: Focus on the n-6 Series

**DOI:** 10.3390/ijms24054567

**Published:** 2023-02-26

**Authors:** Andrea Poli, Carlo Agostoni, Francesco Visioli

**Affiliations:** 1 Nutrition Foundation of Italy, 20124 Milano, Italy; 2 Pediatric Area, Fondazione IRCCS Ca’ Granda—Ospedale Maggiore Policlinico, 20122 Milan, Italy; 3 Department of Clinical Sciences and Community Health, University of Milan, 20122 Milan, Italy; 4 Department of Molecular Medicine, University of Padova, 35121 Padova, Italy; 5 IMDEA-Food, CEI UAM + CSIC, 28049 Madrid, Spain

**Keywords:** linoleic acid, n-6 polyunsaturated fatty acids, human health, cardiovascular disease, neonatal development

## Abstract

Among the polyunsaturated fatty acids (PUFAs), those belonging to the n-3 (or ω3) series, i.e., alpha-linolenic (ALA), eicosapentaenoic (EPA), and docosahexaenoic (DHA) acids have been studied for decades from a pharma-nutritional viewpoint, namely in relation to cardiovascular health. More recent research is focusing on n-6 PUFAs, e.g., linoleic acid (LA), whose levels of consumption are much higher than those of n-3 and that cannot be used “pharmacologically”. Perhaps because of this, the biological actions of n-6 PUFAs have not been investigated in details as those of their n-3 counterparts. However, an increasing body of evidence underscores their healthful actions on the cardiovascular system. Among the critiques to n-6 PUFAs and, particularly, LA there is the fact that they are precursors of pro-inflammatory eicosanoids. Hence, the hypothesis posits that we should reduce their intakes precisely to avoid increasing systemic, low-grade inflammation, i.e., one of the major etiological agents in degenerative diseases. In this narrative review, we address the issue of whether n-6 PUFAs are indeed pro-inflammatory, we discuss the most recent evidence of their role(s) in human health and prognosis, and we conclude that adequate intakes of n-6 fatty acids are associated with better cardiovascular health and child development.

## 1. Introduction

The incidence of many chronic diseases degenerative in nature can be modulated by proper diets [1,2]. Examples are plentiful and include a Mediterranean diet, a Japanese one, and Nordic diet to name a few. Among the major macronutrients, lipids are being paid special attention by researchers because of their ability to modulate several biological processes. Indeed, cell membranes are composed of phospholipids—organized in bilayers—which act as precursors of lipid messengers such as the eicosanoids. Not coincidentally, an inadequate intake of polyunsaturated fatty acids (PUFA) is thought to be one of the most important shortcomings of inadequate diets and is probably more relevant than the reduction of a high dietary intake of saturated fatty acids (SFA) [1].

Among the PUFAs, those belonging to the n-3 (or ω3) series, i.e., alpha-linolenic (ALA), eicosapentaenoic (EPA), and docosahexaenoic (DHA) acids have been studied for decades from a pharma-nutritional viewpoint, namely in relation to cardiovascular health [3].

More recent research is focusing on n-6 PUFAs, e.g., linoleic acid (LA), whose levels of consumption are much higher than those of n-3 and that cannot be used “pharmacologically” [4].

Perhaps because of this, the biological actions of n-6 PUFAs have not been investigated in details as those of their n-3 counterparts. However, an increasing body of evidence underscores their healthful actions on the cardiovascular system. Their involvement in other pathologies, e.g., cancer and neurodegeneration, remains elusive.

Among the critiques to n-6 PUFAs and, particularly, LA there is the fact that they are precursors of pro-inflammatory eicosanoids. Hence, the hypothesis posits that we should reduce their intakes precisely to avoid increasing systemic, low-grade inflammation, i.e., one of the major etiological agents in degenerative diseases.

In this narrative review, we address the issue of whether n-6 PUFAs are indeed pro-inflammatory and we discuss the most recent evidence of their role(s) in human health and prognosis.

## 2. Linoleic Acid Biochemistry

Even though LA is the most abundant PUFA in human diet, it is an essential fatty acid, i.e., it cannot be synthesized by mammals. Its essentiality was first shown by researchers who reported that mice on a fat-free diet developed a deficiency syndrome with neurological and cutaneous symptoms which disappeared following the administration of LA [5]. According to some calculations, humans need approximately 2 g/d of LA to prevent deficiency. This amount can be easily reached when following a healthy diet [6].

After intake, LA can be metabolized to generate PUFA with a longer carbon chain and a greater number of double bonds; among these, AA is the most abundant and physiologically relevant one. It is important to highlight that both n-3 and n-6 fatty acids use the same biochemical pathways, i.e., enzymes for elongation and desaturation. Therefore, one pervasive theory postulates that and excessive intake of n-6 PUFAs would outcompete n-3 fatty acids for elongation and desaturation, in turn creating an unbalance and enriching cell membranes with pro-inflammatory lipid mediators, namely AA.

This theory is correct from a biochemical viewpoint, yet it has been proven over-simplistic when tested in humans. For example, different cell types have different abilities to metabolize LA. One example is that of endothelial cells, where the conversion of LA to AA is rather small and where AA is important to maintain proper vasomotion, e.g., by being the precursor of prostacyclin [7]. Likely, the endothelium requires preformed AA to preserve its intracellular arachidonate stores. Further, the conversion of LA to AA is approximately 40% by THP-1 monocytes and ~10% by HepG2 cells [8]. Astrocytes, yet not neurons, do convert LA to AA, thus supporting the neuronal need of this important fatty acid [9]. Finally, while it is true that elongases and desaturases preferentially use n-3 fatty acids as substrate (followed by n-6 and n-9 fatty acids) [10] their activity depends upon several factors. For example, glucose, insulin, or low-essential fatty acids diets increase the activities, whereas age, adrenaline, glucagon, steroids, or diets rich in cholesterol, oxysterols, and marine oils reduce it. Finally, some drugs affect the aforementioned enzymatic pathways. For example, statins and fibrates promote desaturase activity, yet calcium channel blockers inhibit it [8,10,11]. In brief, the interrelation of fatty acids and elongating and desaturating enzymes is quite complex and not easy to predict merely based on biochemical pathways.

## 3. Metabolic Fate of Linoleic Acid in Organs and Tissues

After fat digestion, i.e., hydrolysis by lipases and/or emulsification by bile salts, LA follows the classic metabolic routes of dietary fat, i.e., it is absorbed by enterocytes from the lumen of the small intestine. Then, LA is re-esterified into a triacylglycerol inside those enterocytes or, to a lesser extent, into phospholipids or cholesteryl esters. The resulting species are subsequently incorporated into chylomicrons and enter the bloodstream.

Once LA penetrates cells, it can be incorporated into cell membranes (as part of the lipid bilayer and mostly in the sn-2 position of phospholipids, TG, and cholesteryl esters) or it can be used for metabolic reactions, according to the type of tissues and their energy requirements. In the circulation, LA is mainly (~30%) concentrated in plasma, where it is transported by LDL and HDL (35 and 30%, respectively), and—to a lesser extent—by platelets and red blood cell membranes (9.3 and 9.7%, respectively). In terms of incorporation into plasma lipids, LA is largely (~50%) esterified in cholesteryl esters, followed by phospholipids (40%) and triglycerides (10%) [12]. In erythrocytes (an important surrogate marker of dietary intake [13,14]), the profile of LA incorporation is similar to that of plasma and platelet lipids [15].

It is noteworthy that LA concentrations in blood are lower in the newborn, where they represent about 4.6% of total fatty acids, and increase during childhood and adulthood up to 17.7% and 18.4% respectively. One potential explanation is that newborns require very high amounts of fatty acids, especially long-chain PUFA, for growth and development (vide infra). Lastly, LA plasma concentrations are often higher in women than in men [16].

In addition to dietary intakes, some genetic variants in genes involved in LA metabolism affect its metabolism and concentrations. For example, genetic variants of the *FADS1* and *FADS2* gene cluster (coding for delta-5 and delta-6 desaturases) result in different tissue levels of specific fatty acids; indeed, up to 28% of the variability in the concentrations of AA is explained by the presence (or absence) of such variants [17,18]. These data add to the notion that “one size does not fit all”: precision nutrition [19] will eventually find the optimal LA intakes of diverse individuals. One example is provided by Schulte et al. [20], who reported that the C-allele of *FADS1* rs174547, related to reduced ability to convert plant-based PUFAs into longer-chain and higher unsaturated fatty acids, is largely absent in African, common in European, and dominant in American populations [20]. Also, the *FADS2* variant rs174570 allele related to lower desaturase activity, is much more frequent in Greenland Inuit than in Chinese or European populations, suggesting a potential human adaptation to varying dietary PUFA sources and paving the road for future precision nutrition guidelines.

## 4. Linoleic Acid and Lower Cardiovascular Disease: Putative Mechanisms of Action

Cardiovascular diseases (CVD) are among the leading cause of mortality worldwide [21]. Several risk factors contribute to their incidence, some of which modifiable via a proper lifestyle and a healthy diet. Recent epidemiological studies single out inadequate or excessive intake of food groups and nutrients in relation to CVD [2,22]. Pertinent to this paper, an inadequate intake of PUFA does contribute to CVD development, likely more than a reduction of high dietary intake of saturated fatty acids, with important heterogeneity among countries [1,23].

Even though the association between high PUFA intake and low CVD has been known for years, the precise mechanisms of action underlying the cardioprotective effects of PUFAs are still to be fully elucidated. Historically, PUFAs in relation to health have been investigated as structural part of cell membranes, where they are incorporated into phospholipids (see above). Indeed, cell membrane phospholipids are precursors of metabolites (lipid mediators) with relevant biologic properties [24].

In terms of PUFAs as related to CVD, n-3 fatty acids are the most studied ones, mostly because they are available as pharmaceutical preparations and because they are bioactive at relatively (i.e., compared to the total amount of dietary fat) low doses. Moreover, their plasma concentrations increase linearly with intake and can, hence, be quite easily modified.

Conversely, n-6 fatty acids are more difficult to investigate from a pharma-nutritional viewpoint because, as mentioned above, they are the most abundant PUFAs in the diet and cannot be administered as pills/tablets/soft gelatin capsules/etc. Only recently, some regulatory bodies such as the EFSA have added n-6 PUFAs to the list of essential nutrients for which a dietary requirement needs to be set [25]. In summary, even though the cardioprotective actions of n-3 fatty acids have not been fully elucidate, there is plenty of in vitro studies addressing their mechanisms of action. Unfortunately, the same does not hold true for their n-6 counterparts.

Yet, epidemiological studies consistently report that an adequate intake of LA is associated with lower plasma concentrations of low-density lipoprotein cholesterol (LDL-C) [26], i.e., the most relevant risk factor for CVD. For example, replacing 5% of the dietary energy derived from saturated fatty acids (SFA) with n-6 PUFA reduces LDL-C by up to 10% (which is very difficult to do in practice because one would need to halve saturates’ intake), in turn greatly reducing CVD risk [27,28]. Moreover, circulating concentrations of LA are inversely associated with incident type 2 diabetes in prospective cohort studies [29].

Experimentally, a diet enriched in n-6 PUFA reduced liver fat and resulted in a modestly improved metabolic status, with no signs of inflammation, compared with a diet enriched in SFA, in cardiometabolically-impaired subjects with abdominal obesity [30].

In brief, ample epidemiological evidence indicates that adequate intakes of LA are associated with better cardiovascular prognosis. Mechanistic studies are scant, but some hypotheses can be formulated.

### 4.1. Effects of LA on Plasma Lipids

Conceivably, the most important contributor to the purported cardioprotective properties of LA is the reduction of blood cholesterol concentration, in particular LDL-C.

One meta-analysis of studies performed in healthy volunteers hosted in metabolic wards reported that the replacement of 5% of dietary energy from complex carbohydrates or SFA with an isocaloric amount of n-6 PUFA leads to a mean reduction of 4.2 mg/dL for plasma total cholesterol (TC) and 15 mg/dL for LDL-C [31]. A systematic review of randomized controlled clinical trials evaluated the effects of replacing 1% of energy from SFA with n-6 PUFA; the results indicate reductions of 0.05 mmol/L (2.1 mg/dL) in LDL-C, 0.01 mmol/L (0.9 mg/dL) in TG and 0.01 mmol/L (0.2 mg/dL) in HDL-C [28]. Another systematic review of 10 dietary intervention studies on 4,280 participants confirmed those conclusions and reported that the highest intakes of n-6 PUFA were associated with a reduction in TC of 13 mg/dL. Yet, there were no major effects on the individual lipid fractions analyzed [32].

Corn oil is very rich in LA and has been used to study the hypocholesterolemic effects of the latter in, e.g., moderately hypercholesterolemic subjects. In one such study, the administration of 54 g/day of corn oil resulted in a more pronounced reduction of TC and LDL-C as compared with other oils, i.e., extra virgin olive or coconut oils [33,34]. Another study confirmed this findings: consumption of 30 g/day of corn oil reduced LDL-C by 13.9%, whereas a 5.8% reduction was obtained by using extra-virgin oil [35].

As far as mechanisms of action are concerned, some biochemical studies have been performed and suggested several pathways through which LA might exert its cholesterol-lowering actions. For example, provision of LA to rodents increased the hepatic expression of LDL receptor (LDLR), which plays a crucial role in the control of plasma cholesterol levels [36]. N-6 PUFA have also been shown to reduce hepatic lipogenesis and concomitantly activate lipid catabolism in vitro, likely via an inhibition of the activity of sterol regulatory element binding protein 1c (SREBP-1c) [37]. Moreover, a study on subjects with abdominal obesity showed that a n-6 PUFA-rich diet reduced plasma levels of proprotein convertase subtilisin/kexin type 9 (PCSK9) [30] compared with SFA intake. However, this finding has not been confirmed by another study, leaving the issue of whether LA modulates this important enzyme unaddressed [38]. Finally, one study undertaken in subjects with both dyslipidemia and insulin resistance, replaced a large proportion of SFA with n-6 PUFA, leading to a lower production and number of LDL particles, mediated by a reduction of the synthesis of apolipoprotein B100 [39].

### 4.2. Effect of LA on Blood Pressure

In addition to keeping LDL-c as low as possible, i.e., “the lower the better” [40], it is also important to maintain blood pressure under control for optimal cardiovascular prevention. Indeed, an appropriate blood pressure does reduce the risk of clinical events associated with arterial hypertension. As always, a healthy diet represents the first approach to prevent hypertension, particularly for the management of subjects that are not yet in need of pharmacological treatment. Several clinical studies (admittedly, mostly of low-moderate quality) have evaluated the effects of PUFA-rich diets on blood pressure. As mentioned above, it is worth reiterating that it is difficult to change the dietary content of n-6 PUFA without simultaneously changing the contribution of other components, e.g., other fatty acids, fiber, (poly)phenols, or minerals, all of which can potentially affect hemodynamic parameters such as blood pressure and flow-mediated dilation [41]. Therefore, published studies just provide estimates of the effect of dietary n-6 PUFA on blood pressure and should be framed in the “replacing one with another” realm of nutrition research.

Some studies did not report any significant effect of increasing intake of n-6 PUFA on the systolic and diastolic pressure [42,43,44,45]. For example, the International Study of Macro-Micronutrients and Blood Pressure, which enrolled 4680 subjects, examined the proportion of dietary LA and found it to be inversely associated with systolic and diastolic blood pressure. Yet, this relationship was not significant [46]. Conversely, a sub-analysis of the “non-intervention” group, i.e., people not a special diet, not taking food supplements or drugs, and free from cardiovascular diseases or diabetes revealed that blood pressure was reduced in a modest yet statistically significant way (−1.4/−0.9 mmHg for each 3.8% more calories from LA) [46]. These data, however, have not been confirmed [47] and a meta-analysis reported no major effects of n-6 PUFA on either systolic or diastolic blood pressure [32].

In summary, it is unlikely that n-6 PUFA and, particularly, LA have important and clinically-relevant effects on blood pressure, ruling out anti-hypertensive effects as one of the major mechanisms underlying LA’s cardioprotective actions and leaving cholesterol control as the major mechanism of action.

## 5. Is There a Trade-Off between LA’s Hypocholesterolemic Action and Inflammation or Oxidative Stress?

As mentioned, adequate intakes of n-6 PUFAs are associated with lower CVD. The mechanisms of actions other than cholesterol control are yet to be fully elucidated, but issue has been raised that the reduction of LDL-c could be offset by increased oxidation and inflammation status. Indeed, all PUFA can easily undergo peroxidation (at least in vitro), depending on their unsaturation degree. Both the production of peroxidized lipids from PUFA, and their complex and often noxious interactions with cell metabolism, including DNA damage and ferroptosis, have been extensively studied [48,49,50].

However, human physiology is more complicated than biochemistry textbooks. For example, fatty acids are present in the human body as esters, which limits their oxidizability; the exposure to oxidizing species, additionally, varies greatly, depending on a large number of factors [51]. In a small intervention study, LA provision (up to 15 g/day) to healthy volunteers did not affect the antioxidant capacity of plasma (a prognostically questionable biomarker), nor the concentrations of markers of DNA damage or lipid peroxidation [52].

Closely related to oxidative stress, inflammation is a major etiological agent of degenerative diseases, such as atherosclerosis, neurodegeneration, and some cancers [53,54]. Lipid mediators such as eicosanoids derive from arachidonic acid (AA) and most of them have pro-inflammatory activities. Hence, the hypothesis posits that, by reducing dietary intakes of n-6 PUFAs, systemic inflammation would be limited. However, the cellular concentrations of AA are tightly regulated and not linearly related to dietary use of LA, as previously mentioned.

Most available data disprove the notion that high intakes of LA lead to systemic inflammation. For example, a cross-sectional sub-study of the BALANCE Program Trial, which evaluated patients with established CVD, analyzed dietary PUFA levels and reported an inverse association with markers of inflammation, including C-reactive protein and interleukin 1β (IL-1β); the increase of 1 g/1000 Kcal in n-6 or n-3 PUFA produced a 8% or 48% reduction of IL-1β, respectively [55]. Similar associations were observed in subjects with insulin resistance, in secondary CV prevention [56]. confirming the results of other studies showing an inverse relationship between circulating levels of n-6 PUFA and inflammatory biomarkers [57,58,59,60]. A meta-analysis concluded that randomized, controlled intervention studies do not provide evidence of a causal relationship between an increased intake of LA and increased concentrations of inflammatory markers [61]. Finally, an epidemiological study by Virtanen et al. [62] reported that the highest quintiles of omega 6 fatty acids were associated with lower CRP concentrations as well as lower cardiovascular, cancer, and all-cause mortality. Interestingly, arachidonic acid or the mainly endogenously produced n-6 PUFAs, gamma-linolenic acid and dihomo-gamma-linolenic acid, were not associated with higher CRP [62].

Even the provision of AA (which is difficult to do because this fatty acid is not easily available) at a relatively high dose did not increase the concentrations of circulating biomarkers of inflammation, at least in healthy volunteers [63,64].

In terms of precision nutrition, the metabolic responses to LA supplementation (including serum hsCRP concentrations) are likely dependent on FADS1 genotype; in part explaining the inter-individual variability reported by some trial [65]. The same holds true for the cholesterol response to LA intake, which is highly dependent on the apoE4 allele [66]. Finally, at least one intervention trial showed that increased intake of LA does not increase circulating concentrations of AA and AA-derived lipid mediators [67].

In summary, it is undisputable that, from a biochemical viewpoint, a LA-derived AA generates pro-inflammatory molecules. However, the available human studies do not confirm such assumption. Possibly, this is due to the aforementioned low in vivo conversion rate of dietary LA to AA; moreover, there is a wide variety of eicosanoids produced by AA, some of which are indeed pro-inflammatory whereas other ones have anti-inflammatory activities [68]. One example is that of wound healing, where LA acts both in the inflammatory phase and in that of tissue repair [69].

Thus, available data from epidemiological and intervention studies suggest that n-6 PUFA levels are not associated with increase concentrations of markers of oxidative stress [52] and are indeed associated with lower circulating levels of inflammatory markers [57,70].

## 6. An Under-Investigated Area: Linoleic Acid during Fetal and Neonatal Development for Cardiovascular Prevention

Because n-6 fatty acids are supposedly pro-inflammatory, some authors propose that their use in the newborn should be limited. However, in the second half of pregnancy, the need for nutrients essential for the development of the nervous system, including essential fatty acids, increases significantly [71]. Both quantity and quality of fatty acids during pregnancy (but also before conception, as fatty acids accumulate in specific deposits and are mobilized on demand) may affect organ and tissue structure and function before and after birth.

In humans, the role of LA in fetal and infant growth is of major importance, mainly as precursor of AA, which is essential for normal neural development, as well as proper growth [72]. The AA synthesis from LA takes place early in the fetus, and may affect the AA status in the brain, where is transported through the blood-brain barrier [73]. In addition to this process, the fetus also depends on the transfer of preformed AA from the mother via the placenta, with progressively higher concentrations of all long-chain PUFA during gestation (biomagnification) [74].

Even in premature infants an inadequate intake of omega-6 may lead to sub-optimal growth [75]. LA is the main fatty acid present during this stage of development in muscle, skin and adipose tissue [76] where it plays major roles in growth processes, barrier mechanisms and metabolic-energy reserves. Some physiological effects (such as the prevention of skin abnormalities) strictly depend on n-6 PUFA and, above all, LA. Its presence in ceramides is crucial for the maintenance of skin permeability and integrity in early stages of extrauterine life, and LA deficiency has been clearly shown to be associated with serious skin lesions [77].

After birth, infants are supplied with either PUFA and long-chain PUFA with breast milk, which contains around 25% LA in plasma total lipids (that is, around 1 g/dL, based on an average fat content of around 4 g/dL) [78].

Infant formulas have been designed in order to approach the functional effects of human milk with both n-3 and n-6 PUFA, since the use of formulas exclusively supplemented with the n-3 in premature infants has been associated with a limitation of growth [75,79]. 

As an essential fatty acid, LA is included in the composition of infant formula, with a minimum content set at 500 mg/100 kcal and a maximum at 1200 mg/100 kcal in the European Community (Regulation EU 2016/127), representing a wide range of levels. An optimal ratio of parental fatty acids (LA and ALA, respectively) to facilitate the endogenous synthesis of long-chain PUFA) through the manipulation of their absolute concentrations, is actually under discussion [80].

In keeping with the above and ruling out pro-inflammatory actions in the brain, Liu et al. [81] recently published an epidemiological study where they reported that dietary sources of n-6 fatty acids were associated with lower risks for cognitive impairment partially via lowering oxidative stress.

## 7. Are Current Nutritional Recommendations in Line with Scientific Evidence?

Vegetable, namely seed oils are rich in n-6 fatty acids, with LA reaching more than 50% of the lipid content. Other relevant sources of LA are nuts, while lower levels are found in whole grains, legumes, meats of non-ruminants, eggs, and dairy products.

Several authors suggest that the dietary intakes of n-6 PUFA are excessive in most of the western world populations and that such intakes would displace n-3 fatty acids from cell membranes. Hence, a highly debated issue concerns the optimal n-3:n-6 ratio. Some investigators suggest that, over the years and due to the increasing use of seed oil in, e.g., bakery or ready-to-eat foods, this ratio became heavily altered in favor of n-6 fatty acids. However, there is no hard scientific evidence that this ratio (both in the diet and in circulating lipids) is a useful concept [82] nor that it is related to cardiovascular health. The majority of lipid experts does not consider this ratio as a useful concept, also because it does not contemplate the absolute quantities of n-3 and n-6 used in the diet [20]. In other words, one can become deficient of essential fatty acids while keeping the aforementioned ratio below putative excess. Indeed, advancements of agricultural procedures as well as altered lifestyles and eating habits did lead to a substantial increase in the use of n-6 PUFA over the last decades or even centuries [83]. Seed oils are becoming more affordable and available [84].

The effect of such increase in n-6 PUFA consumption on human health is still unclear. As mentioned, some authors suggest untoward actions of increasing n-6 use [85]. Yet, a study based on worldwide data conversely reported that n-6 PUFA intake is globally below optimal levels, in theory contributing to the high coronary heart disease (CHD) mortality observed worldwide [23]. The Global Burden of Disease 2017 group [1] for example, points to an insufficient dietary supply of n-6 PUFA as one of the most relevant issues linked to diet-associated CVD.

Because nutritional recommendations and guidelines shape health policies, it is important to analyze and compute both the quantity and quality of dietary fats within the frame of a healthy diet [86].

The optimal limits for total and saturated fat intakes, set by learned societies, usually comprise less than 30–35% and 10% of total energy, respectively. Monounsaturated fats are usually excluded from guidelines, mostly because they are thought to have a neutral impact on cardiovascular risk. The recommended intake for total PUFA, i.e., n-3 and n-6 ranges between 5% and 10% of the energy.

The recommendations for n-6 and, in particular, for LA are less homogeneous and most societies set them between 2.5% and 5% of total energy [25,87] (Table 1). Higher levels (8–10%) likely result in a better control of LDL-c concentrations. Of note, International guidelines dot usually provide information on the dietary sources of n-6 PUFA/LA. [88,89]. As discussed above, no indication is and should be given on the optimal ratio between n-6 and n-3 intake, as underscored by many International Organizations [87,90,91,92,93,94]. The European Food Safety Authority (EFSA) did not set an upper safe level of intake for LA, because of the lack of important toxicity data [25].

## 8. Conclusions

Both dietary intakes and circulating concentrations of n-6 fatty acids inversely correlate with cardiovascular disease risk. The extent and precise nature of such inverse association is yet to be fully elucidated. However, preliminary evidence is strongly suggestive of cholesterol-lowering effects of LA and of some beneficial effects on glucose metabolism.

Albeit quite paradoxical from a mere biochemical viewpoint, accrued evidence suggests that LA might have a beneficial effect on inflammatory parameters, causally correlated with the development of many degenerative diseases and, therefore, likely associated with CVD risk reduction. Indeed, the theoretical competition of n-6 fatty acids with their n-3 counterparts does not appear to hold true in vivo and the Diet Inflammatory Index [95] puts omega 6 fat among the anti-inflammatory food components. For example, Zhuang et al. analyzed 521,120 participants aged 50 to 71 years from the National Institutes of Health-American Association of Retired Persons Diet and Health Study, with 16 years of follow-up, concluded that total mortality is significantly reduced in association with increasing intakes of marine n-3, total n-6, LA, while the n-3:n-6 ratio was irrelevant on the same parameter, in a fully adjusted model [96]. These (and other) data support several conclusions drawn by Stanley et al. [82] and suggest that the n-3:n-6 ratio is not associated with CV risk or mortality. Hence, the widely-used AA:EPA ratio might not be a good proxy for human pro-inflammatory status and both n-3 and n-6 have anti-, rather than pro-inflammatory actions [97].

In conclusion and in agreement with International guidelines (Table 1), increased consumption of PUFA (n-6 and n-3 alike)-rich food is associated with better cardiovascular prognosis [98]. Future studies with adequate power will eventually confirm these data.

## Figures and Tables

**Table 1:** International and National recommendations for the intake of total fats, essential fatty acids, and n-6 fatty acids for the adult general population.

	EU [90]	ITALY [91]	Nordic Countries [92]	France [93]	FAO/WHO [94]	USA [87]
Total lipids (% Energy)	20–35	20–35	25–40	35–40	<30–35	20–35
Total PUFA (% Energy)		5–10	5–10		6–11	
Total EFA (% Energy)			≥3		≥3	
Total n-6 PUFA (% Energy)		4–8				
Linoleic acid (% Energy)	≥4			≥4	≥2.5	5–10

PUFA, polyunsaturated fatty acids; EFA, essential fatty acids.
